# Predicting osteoporosis preventive behaviors in middle-aged and older urban Shanghai residents: a health belief model-based path analysis in a multi-center population study

**DOI:** 10.3389/fpubh.2023.1235251

**Published:** 2023-09-27

**Authors:** Yichen Wang, Chenrui Li, Ruijie Chang, Yongxia Qiao, Yong Cai, Tian Shen

**Affiliations:** ^1^Hongqiao International Institute of Medicine, Tongren Hospital, Shanghai Jiao Tong University School of Medicine, Shanghai, China; ^2^Department of Public Health, Hongqiao International Institute of Medicine, Tongren Hospital, Shanghai Jiao Tong University School of Medicine, Shanghai, China; ^3^School of Public Health, Shanghai Jiao Tong University, Shanghai, China; ^4^Center for Community Health Care, Hospital Development Institute, Shanghai Jiao Tong University, Shanghai, China

**Keywords:** osteoporosis, HBM model, preventive behavior, belief, path analysis

## Abstract

**Background:**

Osteoporosis represents a significant health concern as a widespread metabolic bone condition. In this study, we aim to utilize path analysis to examine the intricate relationships among demographic information, Health Belief Model (HBM) constructs and osteoporosis preventive behavior among Shanghai residents over 40 years of age.

**Methods:**

A multi-center population study was conducted in 20 volunteer communities in Shanghai, China. Out of the 2,000 participants who volunteered, 1,903 completed the field survey.

**Results:**

56.0% of participants were females. Their mean age was 63.64 ± 10.30 years. The self-efficacy score among females (42.27 ± 15.82) was also significantly higher than that among males (40.68 ± 15.20). in the pathway analysis. In the path analysis preventive behaviors were significantly predicted by education (*β* = 0.082, *p* < 0.001), knowledge (*β* = 0.132, *p* < 0.001) and self-efficacy (*β* = 0.392, *p* < 0.001).

**Conclusions:**

This study highlights the importance of gender, education, knowledge and self-efficacy in promoting OP preventive behaviors using the Health Belief Model. The findings emphasize the need for tailored interventions to address the specific needs of different demographic groups.

## 1. Introduction

Osteoporosis (OP) represents a significant health concern as a widespread metabolic bone condition marked by diminished bone mass and disrupted bone structure, resulting in weakened bone integrity and heightened risk of fractures (1). Often referred to as a silent disease, OP typically presents no symptoms until an initial fracture takes place. Clinically significant due to its status as the primary risk factor for fractures, OP's most severe complication involves fractures themselves. These can occur at any bone location, with the vertebrae being the most frequently affected areas and the hip being the most severe part. Osteoporotic hip fractures, in particular, are especially devastating, accounting for up to 5% of total mortality for both men and women (2), and causing 21%−30% of patients to die within a year (3). Additionally, these fractures impose significant financial strain on healthcare systems. In 2010, the economic impact of osteoporotic fractures in 27 EU countries was estimated at €37 billion, with projections indicating a 25% increase by 2025 (4, 5).

The global prevalence of OP has risen sharply alongside the expanding older adult population and the increase of access to investigation tools in recent years. As the population continues to age, the incidence of OP increases dramatically worldwide (6, 7). The overall prevalence of OP among older men and women globally stands at 35.3 and 12.5%, respectively, with the highest prevalence reported among seniors in Asia at 24.3% (8). OP's substantial influence on public health is evident through its associated morbidity, mortality, and healthcare expenses. Consequently, it is crucial for health policy-makers to devise strategies for preventing OP within the older adult population.

Reducing the burden of OP necessitates effective preventative measures. Proven strategies for preventing osteoporotic fractures include sufficient calcium and vitamin D intake, physical activity, early detection, early therapy against osteoporosis and fall prevention techniques (9, 10). However, adherence to these practices is often less than ideal due to various barriers, such as diminished gastrointestinal function, athletic ability, lack of knowledge, perceived low susceptibility, and insufficient social support.

Over the age of 50, the rate of fracture occurrence in different bones, particularly the hip and spine, tends to increase among individuals, considering both genders (11). However, it's important to note that the reduction in bone mineral density (BMD) actually starts around the age of 40 (12). This suggests that interventions for preventing osteoporosis should be initiated as early as possible to delay the onset of osteoporosis and fractures, thereby enhancing the quality of life for the older adult population.

Comprehending the factors that impact preventative behaviors is crucial for devising successful interventions and policies that promote OP prevention and enhance public health outcomes. The Health Belief Model (HBM) is a popular theoretical framework that outlines the connections between individuals' beliefs, self-efficacy, and behaviors concerning health issues (13, 14). Nevertheless, most studies have focused solely on the direct effects of the model constructs on health behaviors, without exploring the intricate relationships among these variables. Furthermore, the sample size in some studies tends to be relatively small (15). It is essential to investigate the mechanisms by which health belief constructs influence health behaviors using a credible sample size.

In this study, we aim to utilize path analysis to examine the intricate relationships among demographic information, the HBM constructs and OP preventative behaviors among Shanghai residents over 40 years of age.

## 2. Methods

### 2.1. Study design

This cross-sectional study, with a descriptive-analytical approach and path analysis, investigated the predictive role of social-demographic characteristics and the HBM components in the osteoporosis preventive behaviors among residents aged 40 and above in Shanghai, China.

### 2.2. Study site

The study took place in the urban region of Shanghai, China, from July to September 2016. As the most populous and developed city in China, Shanghai faces a considerable challenge in addressing its rapidly aging population (16). This made it an appropriate setting for the research, considering that the city has the highest percentage of older residents in the country. Projections indicate that by 2025, the older adult population in Shanghai will surpass six million, with the city's aging rate anticipated to exceed 40% (17).

### 2.3. Study population, sampling size, and procedure

In our study, we used a convenience sampling method across multiple centers to enroll participants. We recruited urban Shanghai residents aged 40 and above from 20 volunteer communities, ensuring a balance between rural and urban regions. With a reported prevalence of osteoporosis in China at 6.46 and 29.13% for men and women aged 50 and older, respectively (18), we assuming a 30% prevalence rate for OP among residents aged 40 and above. With this assumption and the formula *N* = 400*Q*/*p* = 400 ^*^ 0.7/0.3 = 933, we calculated a required sample size of 1,866 people, which was then doubled to account for sampling error. After adding an 8% rejection rate, the total sample size was set at 2,000. Out of the 2,000 participants who volunteered, 1,903 completed the field survey (95.15%).

### 2.4. Ethics

The study received review and approval from the Ethics Committee of the School of Public Health at Shanghai Jiao Tong University before commencement. All participants were given comprehensive information about the study's aims, processes, and potential risks and benefits associated with participation prior to enrollment. Furthermore, at the outset of the questionnaire, participants were required to give written informed consent, confirming their full understanding of the study's implications and voluntary agreement to participate.

### 2.5. Data collection

The study employed a one-to-one interview approach, in which an interviewer asked questions and documented the responses of participants aged 40 years and older. The questionnaire's content was derived from the OP Knowledge Test (OKT) (19) and the OP Self-Efficacy Scale (OSES) (20). Demographic information collected in the study included age, gender, height, weight, education, marital status, employment status (full-time, part-time, unemployed, retired), monthly income, constructs of knowledge and self-efficacy (19, 20). Both scales were translated into Chinese and demonstrated suitability for middle-aged and older in the community, based on their reliability and validity analyses (21, 22).

### 2.6. Measures

#### 2.6.1. Knowledge of OP

OP-related information was measured using the OKT (19) including 15 items, with three possible answers plus “do not know.” Correct answers were given a score of one, while wrong or “do not know” answers scored zero, with the score range of 0–15. Higher scores indicate peoples have access to more knowledge.

#### 2.6.2. Perceived benefits of healthy behavior

The level of perceived benefits was assessed using the OKT including two items, with three possible answers plus “do not know,” reflecting the perception of “a balanced diet can avoid OP” and “regular exercise can avoid OP.” Correct answers were given a score of one, while wrong or “do not know” answers scored zero, with the score range of 0–2.

#### 2.6.3. Perceived threat of unhealthy behavior

The level of perceived threat was assessed using the OKT including nine items, reflecting the perception of “a diet with less diary can cause OP” and “overweight or obese can cause OP.” Correct answers were given a score of one, while wrong or “do not know” answers scored zero, with the score range of 0–2.

#### 2.6.4. Self-efficacy

The level of self-efficacy was assessed using the OSES (20) including 12 items, with 11-point Likert scales (0–10), reflecting the self-efficacy of “choosing the right type of exercise” or “increasing calcium intake,” with a maximum score of 120. A higher score indicated higher self-efficacy.

#### 2.6.5. OP preventive behaviors

A total of six questions are used to present OP preventive behaviors, which divided into three parts: diet, lifestyle, and exercise. Four questions to present diet are “Pay attention to reading the calcium content on the food ingredients label” or “choosing foods with high vitamin D content (e.g., cod liver oil, spinach).” The question to present lifestyle is “adhering to a scientific lifestyle (e.g., no smoking, no alcohol, no strong tea, strong coffee and carbonated beverages, less sugar and salt, and moderate protein intake).” The question to present exercise is “maintaining aerobic exercise (such as brisk walking, aerobics) three or more times a week for 30 min or more each time.” The actual frequency of behaviors within the past 30 days were assessed with 5-point Likert scales (1–5), 1 point for “never” and 5 points for “often.”

### 2.7. Statistical analysis

The database was established using Epidata 3.0. Data analysis was performed using Statistical Program for Social Sciences version 18.0 (SPSS version 20.0) for Windows. The measurement data were presented by mean and standard deviation (SD) or by numbers and percentages (%) depending on the data distribution. The Pearson correlation matrix analysis method was employed to identify correlations between the factors. To examine the hypothetical pathway model, Structural Equation Modeling (SEM) was conducted using Mplus 8.3. SEM compares the proposed model with actual data to determine their relationship. The fit of the pathway model was assessed using the comparative fit index (CFI), root mean square error of approximation (RMSEA), and the maximum likelihood chi-square values/degrees of freedom ratio. A CFI value >0.9 indicates a good fit, while an RMSEA value below 0.05 suggests a good fit, considering model complexity (23). Additionally, a non-significant likelihood ratio chi-square test indicates a good model fit. However, since chi-square is influenced by sample size, a chi-square/degrees of freedom ratio of 5 or less is considered acceptable (23, 24).

## 3. Results

### 3.1. Social-demographic characteristics of the participants

A total of 1,903 community residents completed the survey, with an average age of 63.64 years (SD = 10.30; range: 40–105), and the majority (56.0%) were female. Nearly 90% of participants were married (89.4%) and fewer than 20% had a degree (17.2%), defined as a junior college or college graduate or above. Most participants (76.4%) were retired and 48.3% earned an average personal monthly income of 3,000–6,000 Chinese Yuan (1 USD = 6.85 CNY). OP preventative behavior is associated with the characteristics of age, gender, education, employment and income. After placing these four demographic factors in the multiple linear regression, only gender and education remained significant (Table 1).

**Table 1:** Demographic characteristics their associations with preventive behavior and components of health belief among the participants (*N* = 1,903).

**Characteristic**	***N* (%)**	**Knowledge**	**Perceived benefit**	**Perceived threat**	**Perceived barriers**	**Self-efficacy**	**Preventive behaviors**
**Age**							
40–65	1,085 (57.0)	6.55 ± 2.70	0.71 ± 0.57	3.99 ± 2.53	21.10 ± 10.70	42.67 ± 14.83	17.08 ± 5.34
≥65	818 (43.0)	6.12 ± 2.97	0.71 ± 0.56	3.69 ± 2.48	22.96 ± 11.68	40.12 ± 16.39	16.99 ± 5.22
*P*-value		0.001	0.89	0.01	< 0.001	< 0.001	0.701
**Gender** ^*^							
Male	837 (44.0)	6.41 ± 2.81	0.73 ± 0.58	3.68 ± 2.45	22.67 ± 11.00	40.68 ± 15.20	16.51 ± 5.13
Female	1,066 (56.0)	6.32 ± 2.84	0.70 ± 0.56	4.00 ± 2.55	21.30 ± 11.26	42.27 ± 15.82	17.46 ± 5.37
*P*-value		0.491	0.284	0.005	0.008	0.027	< 0.001
**Education** ^*^							
Primary	302 (15.9)	5.73 ± 2.83	0.74 ± 0.58	3.37 ± 2.50	26.07 ± 11.68	35.70 ± 16.15	15.34 ± 4.74
Middle	679 (35.7)	6.27 ± 2.85	0.71 ± 0.59	3.89 ± 2.43	21.48 ± 11.41	42.54 ± 15.79	17.08 ± 5.19
High	595 (31.3)	6.37 ± 2.93	0.66 ± 0.56	3.87 ± 2.55	21.49 ± 10.75	41.70 ± 15.03	17.10 ± 5.43
Junior	201 (10.6)	7.21 ± 2.37	0.76 ± 0.52	4.19 ± 2.56	19.22 ± 9.45	45.39 ± 13.50	18.34 ± 5.43
University	126 (6.6)	6.98 ± 2.43	0.83 ± 0.55	4.31 ± 2.59	20.40 ± 10.52	43.74 ± 15.02	18.55 ± 5.06
*P*-value		< 0.001	0.014	0.001	< 0.001	< 0.001	< 0.001
**Employment (%)**							
Full-time	327 (17.2)	7.14 ± 2.32	0.76 ± 0.55	4.27 ± 2.47	21.95 ± 10.29	41.58 ± 14.30	17.28 ± 5.04
Part-time	47 (2.5)	6.30 ± 2.46	0.74 ± 0.61	4.21 ± 2.52	23.45 ± 11.05	39.60 ± 15.41	18.09 ± 4.84
Unemployment	75 (3.9)	5.65 ± 3.19	0.49 ± 0.50	3.23 ± 2.68	27.01 ± 12.89	34.03 ± 17.82	15.29 ± 5.14
Retired	1,454 (76.4)	6.23 ± 2.89	0.71 ± 0.57	3.79 ± 2.50	21.57 ± 11.20	42.02 ± 15.63	17.05 ± 5.35
*P*-value		< 0.001	0.004	0.001	< 0.001	< 0.001	0.013
**Income (%)**							
≤ 1,600	245 (12.9)	5.91 ± 2.61	0.74 ± 0.59	3.53 ± 2.52	25.45 ± 12.78	36.07 ± 17.42	15.36 ± 5.10
1,601–3,000	597 (31.4)	6.04 ± 2.92	0.70 ± 0.56	3.67 ± 2.43	22.54 ± 11.12	40.44 ± 15.37	16.95 ± 5.19
3,001–6,000	919 (48.3)	6.56 ± 2.84	0.70 ± 0.58	3.97 ± 2.52	20.88 ± 10.77	43.36 ± 15.14	17.42 ± 5.40
6,001–10,000	112 (5.9)	7.11 ± 2.47	0.74 ± 0.52	4.21 ± 2.64	20.08 ± 9.41	43.51 ± 13.46	17.89 ± 4.74
≥10,000	30 (1.6)	7.63 ± 2.11	0.80 ± 0.61	5.63 ± 2.37	18.03 ± 8.31	47.10 ± 10.50	17.70 ± 4.37
*P*-value		< 0.001	0.712	< 0.001	< 0.001	< 0.001	< 0.001

* Means statistically significant in the multiple linear regression.

#### 3.2. OP related knowledge and perception among participants

Table 1 also presents the analysis of perceived threat score among females (Mean = 4.00, SD = 2.55) was significantly higher than that among males (Mean = 3.68, SD =2.45). The self-efficacy score among females (Mean = 42.27, SD = 15.82) was also significantly higher than that among males (Mean = 40.68, SD =15.20). Table 2 shows correlation matrix of variables of health behavior model. All correlation coefficients were statistically significant. The DW value is 1.92.

**Table 2:** Correlation matrix of the variables.

**Characteristic**	**Knowledge**	**Perceived benefit**	**Perceived threat**	**Self-efficacy**	**Preventive behaviors**
Knowledge	1.000				
Perceived benefit	0.248^*^	1.000			
Perceived threat	0.367^*^	0.410^*^	1.000		
Self-efficacy	0.302^*^	0.103^*^	0.178^*^	1.000	
Preventive behaviors	0.263^*^	0.082^*^	0.173^*^	0.442^*^	1.000

* p < 0.001.

#### 3.3. Path analysis of OP preventative behaviors

**Figure 1:**
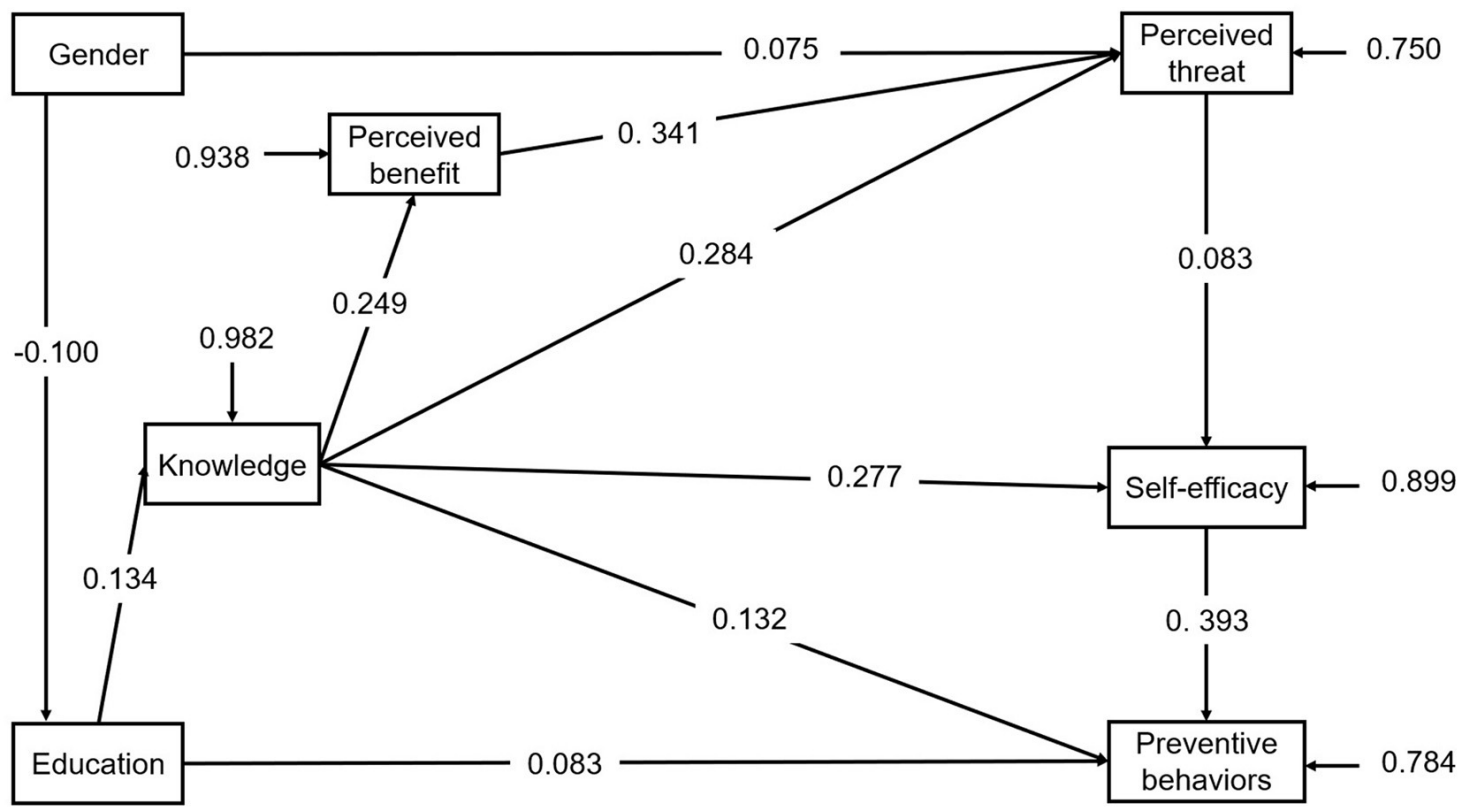
The initial path model for protective behaviors with standardized coefficients. Comparative Fit Index (CFI) = 0.970;Root Mean Square Error of Approximation (RMSEA) = 0.048.

The initial path analysis diagram of the OP preventive behaviors shows in Figure 1. However, the initial model performed poorly for the participants. The model fit indices were as follows: χ^2^ = 53.36, df = 10, *p* < 0.001 thus the χ^2^/df ratio (χ^2^/df = 5.36) exceeded the acceptable range of 5 or less, the Comparative Fit Index (CFI) = 0.970, the Root Mean Square Error of Approximation (RMSEA) = 0.048, the Akaike Information Criterion (AIC) = 57,025.478, Bayesian Information Criterion (BIC) = 57,153.156.

#### 3.4. Modified pathway

**Figure 2:**
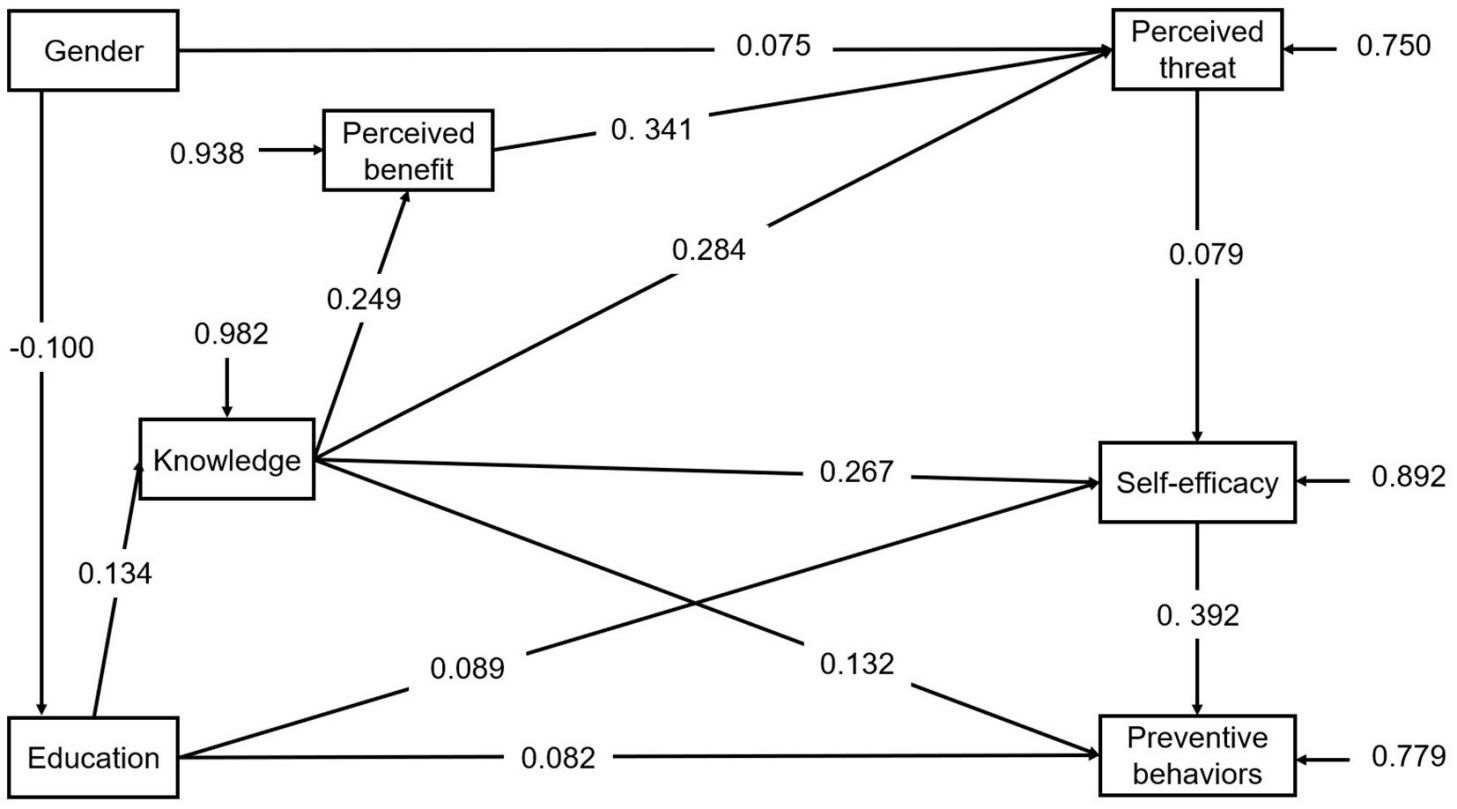
The final path model for protective behaviors with standardized coefficients. Comparative Fit Index (CFI) = 0.980; Root Mean Square Error of Approximation (RMSEA) = 0.040.

To the initial pathway model, we added the supplementary path that knowledge has a direct effect on preventive behaviors. The final pathway model was shown in Figure 2. After modification, the final pathway model performed well for the participants with the acceptable model fit indices: χ^2^= 36.96, df = 9, *p* < 0.001, χ^2^/df = 4.10, CFI = 0.980, RMSEA = 0.040, the Akaike Information Criterion (AIC) = 57,010.914, Bayesian Information Criterion (BIC) = 57,144.142.

As the pathway model predicted, gender of female (*β* = 0.075, *p* < 0.001) had positive effect on perceived threat. Education has a positive effect on knowledge (*β* = 0.134, *p* < 0.001) and preventive behaviors (*β* = 0.082, *p* < 0.001). Knowledge has a positive effect on perceived benefit (*β* = 0.249, *p* < 0.001), perceived threat (*β* = 0.284, *p* < 0.001), perceived self-efficacy (*β* = 0.267, *p* < 0.001), preventive behaviors (*β* = 0.132, *p* < 0.001) respectively. Perceived benefit has a strong positive effect on perceived threat (*β* = 0.341, *p* < 0.001).

The total effect of self-efficacy on preventive behaviors are the highest (*β* = 0.387, *p* < 0.001), followed by that of knowledge on preventive behaviors (*β* = 0.249, *p* < 0.001) as Table 3 shows. The Table 4 shows the *R*-squared value of variable. The modified model explains 21.9% of variance in preventive behaviors.

**Table 3:** Standardized direct and indirect effect on preventive behaviors of modified model.

**Pathway**	**Total effect**	**S.E**.	**Direct effect**	**S.E**.	**Total indirect effect**	**S.E**.
Education	0.152^**^	0.022	0.082^**^	0.020	0.068^**^	0.011
Knowledge	0.24^**^	0.021	0.140^**^	0.021	0.109^**^	0.010
Perceived benefit	0.010^*^	0.003	–	–	0.010^*^	0.003
Perceived threat	0.03^*^	0.009	–	–	0.03^*^	0.009
Self-efficacy	0.387^**^	0.020	0.387^**^	0.020	–	–

* p < 0.01.

** p < 0.001.

**Table 4:** *R*-squared value of variables of modified model.

**Variable**	***R* ^2^**	**S.E**.	***P*-value**
Education	0.010	0.005	0.028
Knowledge	0.018	0.006	0.003
Perceived benefit	0.062	0.011	< 0.001
Perceived threat	0.250	0.017	< 0.001
Self-efficacy	0.101	0.013	< 0.001
Preventive behaviors	0.219	0.017	< 0.001

## 4. Discussion

The prevalence of osteoporosis in China was estimated to be 6.46 and 29.13% respectively, for men and women aged 50 years and older with bone-mineral density (BMD) values decreasing with increasing age (18). Research studies point to a number of risk factors for osteoporosis that are non-modifiable, such as genetic factors. It is well established that the variation in BMD is determined by our genes (25). That doesn't mean we're sitting ducks. It is now proven that some health prompting behaviors may contribute positively to bone health, such as a proper dietary pattern, a good lifestyle and exercise (26, 27).

Older men and women, who often exhibit lower preventive behaviors due to age-related physiological changes and pathological conditions, face a heightened risk of OP (25). Decreasing the incidence of OP in this population could lessen the pain from OP-related fractures and reduce their treatment burdens. To enhance their bone health, lifestyle adjustments and routine check-ups are strongly advised. It is vital to comprehend the factors and mechanisms underlying behavior change. In this study, we employed the Health Belief Model (HBM) to investigate the predictors of OP preventive behaviors among middle-aged and older community members. The HBM elucidates the reasons and circumstances under which individuals will adopt preventive behaviors (28). Our findings highlighted that knowledge is a critical element in altering OP prevention behavior by indirectly affecting HBM factors such as perceived benefits, perceived threats, and self-efficacy, in addition to directly influencing prevention behavior.

Utilizing the HBM to analyze demographic factors associated with OP preventive behavior, it was found that gender, education, employment, and income are connected to preventive behavior. Ultimately, gender and education were included in the model. OP preventative behavior rates were positively correlated with female gender and higher education levels. Individuals with higher education exhibited increased knowledge and were more likely to participate in OP preventive actions. In line with Ishtaya et al.'s findings (29), female participants were more likely to perceive threats from OP, while men displayed lower levels of knowledge and perceived susceptibility (30). The prevalence of OP has been on the rise in recent years, and perceived individual risk of the disease represents a negotiation between gender constructs of OP and various risk factors (31). This suggests that OP prevention programs should actively address gendered and education-based perceptions of OP to promote prevention behaviors across the entire population and reduce future disease incidence.

The correlation matrix reveals significant statistical relationships among beliefs, self-efficacy, and preventive behavior, which is consistent with many other studies (32–34). Although various educational interventions have been developed for preventing or managing OP, their effects on older adults remain inconclusive. A considerable reason for this is that most studies only superficially discuss the relationships among these factors and do not delve deeper into the complex pathways connecting information, beliefs, and self-efficacy. The specific pathways among knowledge, beliefs, and preventive behaviors are often unclear. As a result, we developed a pathway model to establish the action path.

Our study's pathway model demonstrated that preventive behavior is directly influenced by knowledge, education, and self-efficacy, with self –efficacy and knowledge being the most critical determinants of preventive behavior. This finding aligns with Hsieh et al.'s (35) research, which showed a positive correlation between osteoporosis knowledge and self-efficacy for calcium intake and exercise. Although our study partially deviates from previous studies, it confirms that providing knowledge remains a vital factor in OP prevention (36). Our research also established that preventive behaviors are strongly and positively predicted by self-efficacy and beliefs, echoing Hsieh et al.'s results (37). This suggests that enhancing self-efficacy and beliefs may encourage people to adopt more OP prevention behaviors. Health promotion strategies should focus more on self-efficacy and beliefs to foster the adoption of OP preventive behaviors.

Our study discovered a significant impact of education level on the adoption of osteoporosis prevention behaviors. Specifically, individuals with higher education levels are better equipped to acquire and assimilate knowledge, and they exhibit more confidence and awareness in implementing these behaviors. While educational attainment is a factor that cannot be modified, it's possible to tailor interventions and policies to be more focused on less educated groups. This approach can enhance the effectiveness of interventions in preventing osteoporosis.

While our study fills the gap in osteoporosis prevention behavior among middle-aged and older, several limitations should be acknowledged. First, the cross-sectional design precludes the evaluation of self-efficacy changes following enhancements in information and beliefs. Future research could employ longitudinal designs to investigate the causal relationships between these factors and OP preventive behaviors. Second, our study relied on self-administered questionnaires, which may introduce biases in information comprehension; however, having trained investigators present to explain questions to participants may have minimized this potential bias. Third, our study focused on community residents in Shanghai, limiting the generalizability of the findings to all populations or settings. Future research should explore these associations in different regions and cultures to develop a more comprehensive understanding of the factors influencing OP prevention.

## 5. Conclusion

This study highlights the importance of gender, education, knowledge and self-efficacy in promoting OP preventive behaviors using the Health Belief Model. Our findings underscore the importance of reinforcing OP-related beliefs and customizing interventions to address the distinct needs of various demographic groups, such as gender and education. Future research should explore these associations across various regions and cultures, employing longitudinal designs to improve public health outcomes related to osteoporosis prevention.

## Data availability statement

The raw data supporting the conclusions of this article will be made available by the authors, without undue reservation.

## Ethics statement

The studies involving humans were approved by the Ethics Committee of the School of Public Health at Shanghai Jiao Tong University. The studies were conducted in accordance with the local legislation and institutional requirements. Written informed consent for participation was required from the participants or the participants' legal guardians/next of kin in accordance with the national legislation and institutional requirements.

## Author contributions

Study design: YC and TS. Data collection: YW, CL, and RC. Data curation: YW, RC, and YQ. Writing original draft and formal analysis: YW and CL. Writing—review and editing: YQ, YC, and TS. All authors have read and agreed to the published version of the manuscript.
